# Predicted Functional RNAs within Coding Regions Constrain Evolutionary Rates of Yeast Proteins

**DOI:** 10.1371/journal.pone.0001559

**Published:** 2008-02-13

**Authors:** Charles D. Warden, Seong-Ho Kim, Soojin V. Yi

**Affiliations:** 1 School of Biology, Georgia Institute of Technology, Atlanta, Georgia, United States of America; 2 Division of Biostatistics, Indiana University School of Medicine, Indianapolis, Indiana, United States of America; Indiana University, United States of America

## Abstract

Functional RNAs (fRNAs) are being recognized as an important regulatory component in biological processes. Interestingly, recent computational studies suggest that the number and biological significance of functional RNAs *within coding regions* (coding fRNAs) may have been underestimated. We hypothesized that such coding fRNAs will impose additional constraint on sequence evolution because the DNA primary sequence has to *simultaneously* code for functional RNA secondary structures on the messenger RNA in addition to the amino acid codons for the protein sequence. To test this prediction, we first utilized computational methods to predict conserved fRNA secondary structures within multiple species alignments of S*accharomyces sensu strico* genomes. We predict that as much as 5% of the genes in the yeast genome contain at least one functional RNA secondary structure within their protein-coding region. We then analyzed the impact of coding fRNAs on the evolutionary rate of protein-coding genes because a decrease in evolutionary rate implies constraint due to biological functionality. We found that our predicted coding fRNAs have a significant influence on evolutionary rates (especially at synonymous sites), independent of other functional measures. Thus, coding fRNA may play a role on sequence evolution. Given that coding regions of humans and flies contain many more predicted coding fRNAs than yeast, the impact of coding fRNAs on sequence evolution may be substantial in genomes of higher eukaryotes.

## Introduction

There are numerous exceptions to the “standard” flow of genetic information depicted in the central dogma of molecular biology. For example, some genes code for “non-coding” RNA (ncRNA) that are never translated. Such non-coding RNAs play important roles in vital biological processes, especially in the regulation of gene expression [1], [2]. Here, we use the term functional RNA (fRNA) to refer to both ncRNA and conserved fRNA secondary structures within coding regions.

Interestingly, recent computational studies have predicted a large number of functional RNA secondary structures *within* protein-coding regions (referred to as ‘coding fRNA’) in vertebrates [3], yeast [4], and flies [5]. There are already some known examples of coding fRNAs playing significant biological roles in higher eukaryotes, such as in programmed frameshifting [6], A-to-I RNA editing [7], and selenocysteine insertion at stop codon sites [8]. However, the predicted *abundance* of coding fRNAs is surprising. If the majority of these computational predictions are accurate, the presence of coding fRNAs will play an important role in molecular evolution of protein sequences.

Specifically, we hypothesize that the presence of coding fRNAs will impose additional evolutionary constraint on coding sequences because coding fRNAs require the DNA primary sequence to *simultaneously* code for conserved secondary structures in addition to the amino acid sequence. As far as we are aware, the influence of coding fRNAs on evolutionary rates has not yet been explored.

In this study, we investigate the distribution and evolutionary impact of predicted coding fRNAs in *Saccharomyces cerevisiae*. Although there are relatively few known fRNAs in the yeast genome, studying yeast has several advantages. First, there are many sequenced yeast genomes available, enabling comparative studies. The divergence among the *Saccharomyces sensu stricto* genomes is comparable to that among the eight vertebrate genomes used to search for fRNAs in an earlier study [3]. Second, advances in yeast functional genomics have provided a wealth of other functional genomics data to be used in evolutionary analysis [9]–[11]. Third, yeasts are more conducive to experimental manipulation, so the biological function of a predicted fRNA can be learned more easily, compared to vertebrates. For example, an fRNA may play a tissue- and developmental stage- specific role in humans and cannot be easily validated by experimental methods. Thus, our search for coding fRNAs in yeast complements similar computational searches in higher eukaryotes and provides a valuable set of coding fRNA candidates for future experimental studies.

We found that as much as 5% of the genes in the yeast genome may contain at least one coding fRNA. These predicted coding fRNAs tend to constrain evolutionary rates in protein coding regions. In particular, evolutionary rates at synonymous sites were strongly affected by the proportion of predicted coding fRNAs within a gene. This relationship was independent of other functional variables known to affect protein evolutionary rates in yeast. Thus, the yeast genome may contain a considerable number of *coding* functional RNAs that decrease protein evolutionary rates.

## Results

### Yeast genome harbors a substantial number of coding fRNAs

We used several filtering steps while combining two prediction methods to assess the distribution of functional RNA secondary structures within genomes of the *Saccharomyces sensu stricto* clade. The first method, implemented in the EvoFold program, uses a phylogenetic stochastic context free grammar (phylo-SCFG) model that identifies fRNA based upon substitutions that maintain a conserved secondary structure among nucleotide sequences in a multiple species alignment [3]. The second prediction method, implemented in the RNAz program, utilizes information on both conserved secondary structure and thermodynamic stability to identify RNA secondary structures in multiple sequence alignments [12]. We believe our methodology (outlined below) has produced a stringently defined set of potential fRNAs that should be useful in determining targets of future investigation. For further details, please refer to the Methods section and Supplementary Text S1.

We first determined ‘optimal’ sets of comparative alignments by maximizing the number of known ncRNAs, serving as positive controls, recovered by different prediction conditions (Text S1). RNAz and EvoFold exhibit different sensitivity in this positive control test (Table S1), reflecting the fundamental differences in their algorithms. From this analysis, we determined that data set with the best predictive power was the set of EvoFold predictions produced by the 5-species alignment (with an FPS value greater than 0, see below) that were independently verified by the RNAz predictions made using the 6-species alignment (with P-value of 0.9, see below). The number of folds predicted by different methods is shown in Figure S1.

The significance of a predicted fRNA from the EvoFold program was determined by a folding potential score (FPS). FPS is a length normalized likelihood-ratio score and is defined as follows: FPS = log (P(x|φ_fRNA_)/P(x|φ_bg_))/*l*, where P(x|φ_fRNA_) refers to the probability that a sequence fits an fRNA structural model, P(x|φ_bg_) refers to the probability that the sequence fits the background model (i.e. no-fRNA structure model), and *l* refers to the length of the fold (defined by the outermost basepair of a fRNA structure) [3]. We required all folds in the final dataset to have an FPS greater than 0. Requiring a higher cutoff value for the FPS score does not substantially improve the accuracy of our dataset, since it did not increase the recovery of positive controls (results not shown). The error rate of the phylo-SCFG method in EvoFold is predicted to be substantial (around 60%), even though it is difficult to determine the precise false positive rate for these predictions [3].

Next, we chose a subset of fRNAs that were independently verified by EvoFold and RNAz predictions. The RNAz program uses a machine learning technique to produce p-values based on estimated false positive rates [12]. For the set of RNAz predictions, we chose a cutoff P-value of 0.9, which corresponds roughly to a 1% false positive rate according to the RNAz authors [12]. In comparison, a cutoff P-value of 0.5 corresponds to 4% false positive rates.

Furthermore, we removed EvoFold predictions that were shorter than 10 nucleotides because the vast majority of predictions that were less than 10 nucleotides were not likely to form a stable RNA secondary structure.

Following these four steps, we identified 919 predicted fRNAs. When compared to the maximum number of folds that could be predicted for either of these methods (using the 5-species alignment for EvoFold and the 4-species alignment for RNAz), our pipeline for reducing false positives resulted in a 55.5% reduction of EvoFold predictions and an 85.4% reduction in RNAz predictions (Figure S1).

**Figure 1:**
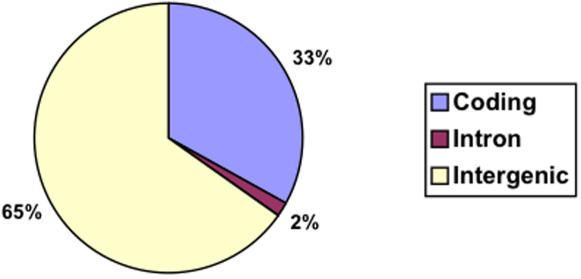
Substantial Proportion of Predicted fRNAs within Coding Regions.

The genomic distribution of these folds is shown in Figure 1. The majority of fRNAs were predicted in intergenic regions. Nevertheless, a considerable proportion (33%) of the total fRNAs was found within protein coding regions. Overall, 272 genes were found to contain at least one coding fRNA. Given that there are approximately 6000 genes in the yeast genome, our results predict that as much as 5% of the yeast proteome may encode at least one coding fRNA.

For our functional analyses, we further restricted our data to only use well-curated genes across different yeast genomes (see Methods). For example, we removed genes with introns, because exon/intron boundaries may not be conserved in different yeast genomes. This procedure left a set of 169 genes. We performed two additional analyses to detect potential false positives. First, we only chose coding fRNAs with negative free energy, which is the minimum thermodynamic requirement to expect coding fRNAs could fold *in vivo*, resulting in 143 coding fRNAs considered for functional analysis (see Methods). Note that results obtained using data without undergoing the last step were qualitatively similar to those obtained using the most restricted data. Second, we used a codon shuffling approach, which also led to similar results (see Methods and Text S1).

The average length for a coding fRNA considered for functional analysis (see Methods) is 22.5±10.3 bps. Coding fRNAs tend to be evenly distributed within coding regions (the average relative position for a coding fRNA is 0.51±0.29 of the length of the coding region).

### Under-representation of coding fRNAs in yeast compared to vertebrates and *Drosophila*

We found that EvoFold had a greater propensity to predict coding fRNAs in vertebrates than in yeast. A previous study for conserved fRNAs in the human genome using the EvoFold program found that 23% of the predicted fRNAs were found within coding regions [3]. For comparison, only 18% of the comparative data set used in this study was coding regions (as measured by the proportion of phastCons elements found within coding regions) [13]. In contrast, we found only 33% of fRNAs in coding regions of yeast, which contain 86.1% of the phastCons elements [13]. Another way to understand this comparison is to note that 303 coding fRNAs were found in 65,348 phastCons CDS blocks in yeast while 12736 coding fRNAs were found in 23,580 phastCons CDS blocks in vertebrates [3], [13]. Thus, in terms of the proportion of coding fRNAs to phastCons CDS blocks, coding fRNAs are about 10 times more likely to be found in vertebrates than yeast.

It should be noted that Pedersen et al. [3] use a slightly different method of defining the fold location for a given fRNA secondary structure, and the phastCons elements was defined across a slightly more diverged multi-species alignment for yeast (longest unconserved divergence = 1.290, 7 species compared) than for vertebrates (longest unconserved divergence = 1.198, 5 species compared) [13]. Still, the large difference in the abundance of coding fRNAs in vertebrates and yeast warrants future investigations of the role of coding fRNA in higher eukaryotes.

A recent study also revealed that a significant number of coding fRNAs reside in *Drosophila* genomes, using the EvoFold program [5]. The distribution of phastCons elements in Drosophila is roughly similar to the phastCons distribution for vertebrates [13]. Thus, it appears that coding fRNAs are less abundant in yeast compared to humans and *Drosophila*. It would be informative to conduct functional genomic analysis similar to that described in this paper on recently discovered coding fRNA predictions in higher eukaryotes to determine the role of fRNAs on coding sequence evolution.

### Genes with coding fRNAs enriched with specific ontology annotations

**Figure 2:**
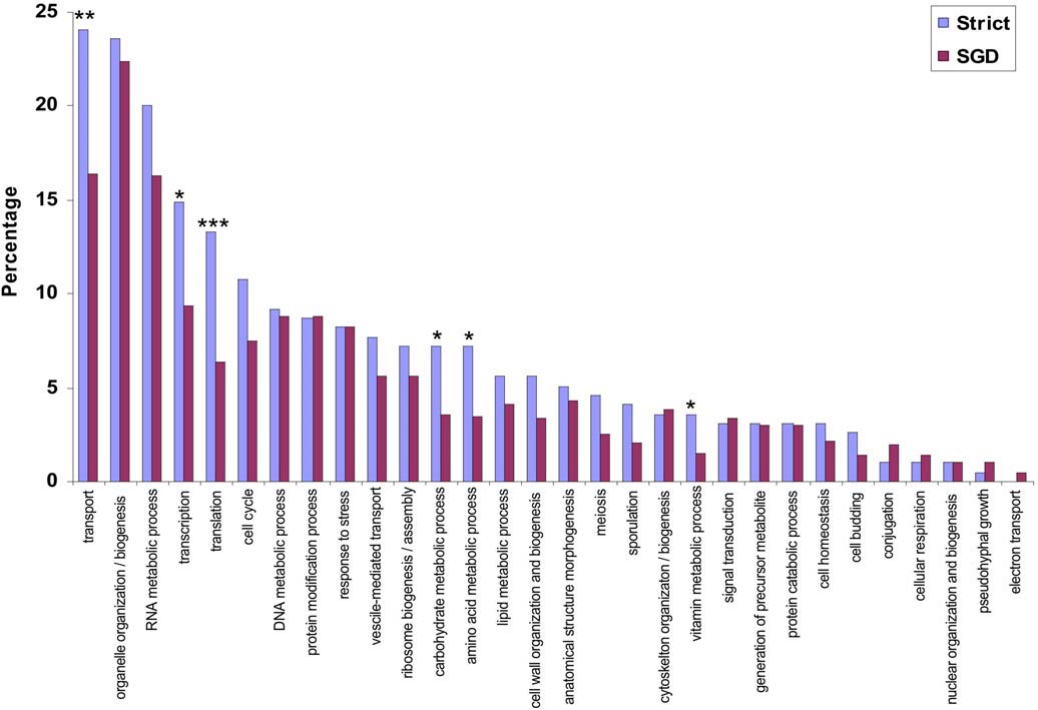
Distribution of GO Annotations in Strictly Defined Dataset. This figure shows the distribution of GO annotations for the set of genes containing at least one predicted fRNA fold (‘strict’) compared to the background set of genes in the yeast genome as annotated in the SGD database (‘SGD’). Some GO annotations have been abbreviated for the interest of space limitation. p-values: * = 0.05, ** = 0.01, *** = 0.001.

We analyzed the distribution of GO annotations for genes containing at least one coding fRNA, to test for possible sources of bias in the dataset and to determine whether genes containing coding fRNAs otherwise tend to be enriched with any particular biological functions. We compared the distribution of GO annotations in our dataset with that in the whole yeast genome and tested for significant deviations (Methods). We found that genes containing coding fRNAs tend to be enriched with the following GO categories: various metabolic processes (amino acid (GO ID: 6519), carbohydrate (GO ID: 5975), and vitamin (GO ID: 6766)), transcription (GO ID: 6350), translation (GO ID: 6412), and transport (GO ID: 6810) (Figure 2). Enrichment with ribosomal genes can be problematic because of some of the unique characteristics associated with these generally well-conserved proteins [14]–[17].

More specifically, the genes associated with translation (GO ID: 6412) have significantly greater values of fRNA coverage and significantly smaller values of evolutionary divergence than the set of all genes in the strictly defined dataset (Wilcoxon rank sum test, p-value<0.05, data not shown). Due to this concern, ribosomal genes (GO ID: 6412 translation) were removed for the statistical analysis described in the following section.

### Predicted coding fRNAs significantly constrain evolutionary rates

Here, we investigate whether the predicted coding fRNAs from yeast genomes are likely to be biologically relevant. If coding fRNAs serve a biological function, then the presence of coding fRNAs should constrain evolutionary rates because of the added constraint to conserve a fRNA sequence on the messenger RNA. Thus, evolutionary rates of genes harboring fRNA should be reduced more than expected based upon other known functional factors. We specifically test this prediction.

Although we employed several filtering steps to identify likely fRNAs, we took additional cautions to remove false positives before evolutionary analysis (see Methods). We note that all the results presented here were qualitatively similar when we performed analyses without this last step or when we used a different approach to eliminate potential false positives (Text S1).

For our functional genomic analysis, we define a new variable, percent ‘fRNA coverage,’ which is the length of the coding region for a gene overlapping a predicted fRNA divided by the length of that gene. First, we assessed how fRNA coverage impacts evolutionary rates. After removing false positives for folds that were not thermodynamically stable, we found that fRNA coverage is negatively correlated with divergence at both nonsynonymous (Pearson's *ρ* = −0.235, *P*<0.05, sample size = 81) and synonymous sites (*ρ* = −0.497, *P* = 0.005). All variables are log transformed to approximate normality. Non-parametric correlation tests provided similar results (data not shown). In order to correct for the impact of codon usage bias on evolutionary rates, we also calculated an adjusted value for synonymous site divergence, dS′ [18]. We found that there are significant negative correlations with dS′. Thus, coding regions with a greater proportion of sequence overlapping coding fRNAs evolve more slowly.

However, we need to ensure that the association between predicted fRNA coverage and evolutionary rate is not due to the influence of a third secondary factor (or set of secondary factors) because many functional variables are known to affect protein evolutionary rates of yeast. Several recent studies emphasize the importance of proper statistical methods to assess independent effects of specific variables of interest [9]–[11], [17]. For example, Wall et al. [10] used partial correlation analysis to show that gene expression and gene dispensability have significant, independent impacts on evolutionary rates. Drummond et al. [9] used principal component regression analysis to conclude that indicators related to gene expression (gene expression, CAI, and protein abundance) are the dominant determinants of evolutionary rates in yeast. We analyzed the impact of fRNA on evolutionary sequence divergence, while controlling for other factors, using both the partial correlation and the principal component regression methods.

We did not consider gene length as a variable in our multivariate analysis because gene length and fRNA coverage are not independent variables (due to the way the variable fRNA coverage is defined: see above). Because gene length and evolutionary rates are positively correlated [11], [19] and fRNA coverage and gene length are negatively correlated, it is important to show that the impact of fRNA coverage on evolutionary rate is still significant when controlling for the influence of gene length. To address this concern, we conducted partial correlation between fRNA coverage and evolutionary rates while controlling for gene length. Significant correlation between fRNA coverage and evolutionary rates remained after this step (Table S2). In addition, we compared the amount of variance in our data that can be explained by fRNA coverage versus to that by 1/(gene length). We observe that fRNA coverage can explain greater amount of variance than 1/(gene length), for all measures of evolutionary rates (Table S2). Thus, fRNA coverage appears as a measure that is more robust than either coding fRNA length or gene length alone.

We considered the following seven functional variables that are known to be important determinants of yeast evolutionary rates: gene expression, CAI, gene dispensability, degree, centrality, mRNA half-life, and fRNA coverage [9], [11]. Among our final data set, there are only 25 genes with all seven variables defined, and only 22 genes after removing genes with negative minimum free energy (*mfe*).

Because analyses of such a small data set may be strongly influenced by stochastic effects, we removed degree, centrality and mRNA half-life from our analyses presented in the main text. It has been shown in earlier studies that network variables such as degree and centrality tend to have minor effects on yeast evolutionary rates [9], [11], [17], and mRNA half-life is not often considered as an important determinant of yeast evolutionary rates. This step allowed us to have a moderate sample size (73 genes).

Table 1 presents Pearson's correlations and partial correlations for genes with negative *mfe*. There is a negative correlation between fRNA coverage and all five measures of evolutionary rates. When all other functional measures are considered for partial correlation analysis, fRNA coverage appears to affect dS′ most significantly. We found similar results with additional functional variables (sample size = 22, Table S3), across a shorter evolutionary timescale (sample size = 128, Table S4). Thus, partial correlation analysis reveals that fRNA coverage imposes a significant constraint on sequence evolution, especially on synonymous sites after correcting for the effect of codon usage bias.

**Table 1:** Correlation and partial correlations show coding fRNAs decrease evolutionary rates (genes with negative *mfe*).

	dN	dS	dS′	dN/dS	dN/dS′
Gene Expression	−0.163 (0.583****)	−0.322 (−0.735***)	−0.203^#^ (−0.237*)	−0.062 (−0.360**)	−0.135 (−0.567****)
CAI	−0.376*** (−0.620****)	−0.514*** (−0.762****)	0.206 (−0.015)	−0.211^#^ (−0.391***)	−0.404*** (−0.632***)
Dispensability	0.293* (0.370**)	−0.170 (0.294*)	0.160 (0.223^#^)	0.233* (0.300**)	0.275* (0.350**)
**fRNA Coverage**	−0.089 (−0.235*)	−0.183 (−0.311**)	−0.334** (−0.409***)	−0.033 (−0.139)	−0.040 (−0.191)

Note: Pearson Correlations are shown in parenthesis below partial correlation in the above table. For above dataset, ribosomal genes are removed and all other factors are considered for partial correlation analysis. Sample size is 73 genes. Significant correlations with fRNA coverage are shown in bold; p-values: # = 0.1, * = 0.05, ** = 0.01, *** = 0.001, **** = 10^−4^.

The relative impact of fRNA coverage on evolutionary rate observed from partial correlation analysis is also corroborated by results from principal component regression analysis (Table 2). The results in table 2 show that principal components 1 and 4 are related to gene expression while principal components 2 and 3 are influenced by fRNA coverage. As seen previously [9], [11], components associated with gene expression explain a significant percent of the variance in the dataset. Interestingly, the principal component 2, which has a large contribution of fRNA coverage, has a strong influence on dS and dS′.

**Table 2 pone-0001559-t002:** Principal component regression reveals coding fRNAs have significant influence on evolutionary divergence (genes with negative *mfe*).

	Principal Components					
	1	2	3	4	All	
Component Composition:^1^						
Gene Expression	**0.425**	0.046	0.006	**0.523**		
CAI	**0.375**	0.171	0.000	**0.454**		
Gene Dispensability	0.101	**0.310**	**0.587**	0.002		
**fRNA Coverage**	0.099	**0.473**	**0.407**	0.021		
Percent Variance Explained:^2^						
dN	**44.48**	0.15	1.62	1.03	**47.28**	
dS	**66.15**	1.52	0.06	0.33	**68.06**	
dS′	**5.64**	**13.01**	1.74	2.90	**23.29**	
dN/dS	**17.60**	0.01	2.43	0.85	**20.88**	
dN/dS′	**42.61**	0.70	2.09	1.57	**46.97**	

1 Numbers in bold correspond to predictors that contribute at least 20% to indicated component.

2 Using information from regression analysis, underlined font means p-values<0.1; bold font means p-value<0.05.

Sample size is 73 genes. Results are similar when considering divergence across a shorter timescale and additional functional variables (see Tables S6,S7).

We also present results obtained after identifying and removing potential false positives using a codon shuffling method (Methods). The sample size is 55 genes. Partial correlation (Table 3) and principal component regression analysis (Table 4) show that the fRNA coverage is generally negatively correlated with evolutionary rates, and the effect is the most pronounced for dS′. In particular, in Table 4 we can see that the effect of fRNA coverage and gene dispensability are separated into components 2 and 3 respectively, and that the component 2 (which mostly represents the effect of fRNA coverage) has a clear effect on dS′. Results are also similar when principal component regression analysis is applied to evolutionary rates when considering additional functional variables (Table S5) and across a shorter timescale (Table S6). Thus, fRNA coverage has a significant, independent impact on evolutionary rates, especially at synonymous sites.

**Table 3 pone-0001559-t003:** Correlations and Partial Correlations using Pearson Correlations on Genes with EFP>0.

	dN	dS	dS′	dN/dS	dN/dS′
Gene Expression	0.035 (−0.430**)	−0.260^#^ (−0.683****)	−0.159 (−0.119)	0.112 (−0.152)	0.057 (−0.414**)
CAI	−0.451*** (−0.563****)	−0.551**** (−0.737****)	0.256^#^ (0.149)	−0.255* (−0.274)	−0.477*** (−0.581****)
Dispensability	0.288* (0.334*)	0.179 (−0.282*)	0.109 (0.143)	0.215 (0.235)	0.273* (0.315)
**fRNA Coverage**	−0.151 (−0.166)	−0.221 (−0.251^#^)	−0.356** (−0.412**)	−0.072 (−0.066)	−0.098 (−0.114)

Note: Pearson Correlations are shown in parenthesis below partial correlation in the above table. For above dataset, ribosomal genes are removed and all other factors are considered for partial correlation analysis. Sample size is 55 genes. Significant correlations with fRNA coverage are shown in bold; p-values: # = 0.1, * = 0.05, ** = 0.01, *** = 0.001, **** = 10^−4^.

**Table 4:** Results of Principal Component Regression Analyses for Genes with EFP>0.

	Principal Components					
	1	2	3	4	All	
Component Composition:^1^						
Gene Expression	**0.460**	0.002	0.029	**0.508**		
CAI	**0.399**	0.125	0.030	**0.445**		
Gene Dispensability	0.111	0.102	**0.780**	0.007		
**fRNA Coverage**	0.029	**0.770**	0.161	0.040		
Percent Variance Explained:^2^						
dN	**30.24**	0.09	0.95	**7.44**	**38.71**	
dS	**61.72**	0.05	1.11	1.53	**64.4**	
dS′	0.08	**20.86**	0.20	2.23	**23.38**	
dN/dS	5.11	0.05	2.49	5.76	13.41	
dN/dS′	**29.62**	0.78	1.06	**8.49**	**39.96**	

1 Numbers in bold correspond to predictors that contribute at least 20% to indicated component.

2 Using information from regression analysis, underlined font means p-values<0.1; bold font means p-value<0.05.

Sample size is 55 genes.

## Discussion

In this study, we demonstrated that there are a substantial number of predicted coding fRNAs in the yeast genome (as much as ∼5% of the protein-coding genes) and that these predicted fRNAs seem to play a biologically significant role (based upon statistical analysis of evolutionary rates). More specifically, genes containing a larger proportion of fRNAs evolve significantly more slowly at synonymous sites, independent of codon usage bias and effects of other functional variables (see Tables 1–4).

Coding fRNAs may have a stronger effect on evolutionary rates at synonymous sites than at nonsynonymous sites, because there are many more sources of functional constraint for nonsynonymous sites, thus requiring a survey with greater statistical power to understand the more subtle influences of coding fRNA on nonsynonymous rates. It is also interesting that coding fRNAs have a relatively greater influence on synonymous site evolution because synonymous sites are traditionally considered to evolve at a neutral rate, and we show that predicted coding fRNAs may be a significant source of non-neutral evolution at synonymous sites. Below we discuss the limits of computational predictions, and factors that could have influenced our statistical analyses and the conclusions on evolutionary impacts of coding fRNAs.

### Determining false positive rates for predicted fRNAs

Although it is difficult to gauge the statistical power of this search for coding fRNAs because there are not many known coding fRNAs in yeast, there is promising evidence that our final set of predicted fRNAs has successfully recovered biologically relevant fRNA secondary structures. For example, HAC1 is a well-studied gene in yeast that undergoes *non-spliceosomal splicing* for dual-coding regions, and the mRNA for this gene is known to require conserved mRNA secondary structures in order to undergo alternative splicing [20], [21]. A stringently defined coding fRNA was recovered within HAC1 (although it should be noted that this gene was excluded from statistical functional analysis because it contains an intron). It is interesting to note that many dual-coding genes in higher eukaryotes (*GNAS1, XBP1, INK4a*, and *ADCY8*) discussed in a recent study [22] also contain at least one EvoFold prediction of a coding fRNA [3]. Therefore, the results of this and similar studies may help explain the splicing mechanisms for dual-coding regions and other exciting biological functions associated with coding fRNAs.

Some previous studies have discovered novel ncRNAs in yeast. For example, one earlier study used the QRNA program to produce a list of ∼100 ncRNA candidate genes [23]. More recently, a study identified a number of novel candidate coding and non-coding fRNAs in yeast [4]. To our surprise, none of our stringently defined coding fRNAs overlap with predictions from the most stringently defined set of coding fRNAs in Steigele et al. [4].

This observation is a poignant reminder that the current fRNA prediction programs and false-positive tests suffer from a large and essentially unknown error rates, and different computational methods likely to respond to different signals and/or categories of fRNAs. Indeed, it has been noted that in vertebrates, the predictions by the RNAz and EvolFold have less than 10% overlap [24].

Another source of discrepancy between our results and those in Steigele et al. [4] is that in the latter the authors used an RNAz scoring measure that placed greater emphasis on conserved covariance between sites, whereas average thermodynamic stability between species was the dominant factor determining which RNAz predictions were defined in our dataset. Nevertheless, given that we used commonly used algorithms (EvoFold [3], [5], [25]–[29], and RNAz [4], [12], [28]–[33]) and that we used several filtering steps, including two different methods to exclude potential false positives (Methods), we consider our results to have strong computational support.

Ultimately, the only way to determine true false positive rates is experimental validation. Thus, our results should provide a valuable complement to this earlier study and provide experimental scientists with a new list of candidate coding fRNAs. Our results should be also helpful to better evaluate computational methods to predict fRNAs.

### Prediction methods are not biased by evolutionary constraint

Although there is clearly a significant negative correlation between fRNA coverage and evolutionary rates, it is necessary to show that the correlation between percent fRNA coverage and evolutionary rates is not due to a bias in prediction methods. The RNAz program is not known to have any specific bias towards predicting false positives [12]. In fact, even though the RNAz program was designed to search for *non-coding RNAs*, it predicted a *larger* proportion of coding fRNAs than EvoFold (Text S1). In comparison, EvoFold requires moderately well conserved multiple species alignment to successfully predict fRNA secondary structures. In particular, EvoFold's measure of significance for folds, FPS (see Results section), has a bias towards ranking highly-conserved, short fRNAs with a high FPS [3]. Indeed, we observed a negative correlation between FPS and synonymous and nonsynonymous rates in our original predicted fRNAs. However, these correlations were mainly caused by ribosomal genes. When we removed ribosomal genes from our data set, FPS was no longer significantly correlated with evolutionary rates. Furthermore, there is no significant difference in the average value for evolutionary rates in genes with short versus long fRNAs (see Table S7). Thus, it is unlikely that our analysis is biased due to spurious predictions of multiple and/or short coding fRNAs within conserved genes.

### Conclusions

Our results indicate that the presence of coding fRNAs constrain evolutionary rates of yeast proteins. The list of coding fRNAs presented in this study should warrant future experimental validation. Since coding fRNAs are likely to be more prevalent in genomes of higher eukaryotes including human and *Drosophila*, the impact of coding fRNA on sequence evolution in those species is likely to be substantial. Overall, this study suggests that the evolutionary impact of coding fRNAs may have been underestimated.

## Methods

### Functional RNA predictions

We use the EvoFold and RNAz algorithms to screen the Multiz alignment for *Saccharomyces sensu stricto* species for functional RNA secondary structures [3], [12], [34]. EvoFold is a program that uses comparative genomic analysis to identify conserved fRNAs based upon compensatory substitutions required to maintain a particular RNA secondary structure [3]. In contrast, the RNAz program uses comparative genomic analysis to compare independently predicted RNA secondary structures for a multiple species alignment based upon thermodynamic predictions form each species' primary sequence [12]. We required our fRNAs to be independently verified by both of these very different methods (in addition to other strict requirements – see “Calculation of Nonsynonymous and Synonymous Divergence” section).

Screening for functional RNAs was conducted using EvoFold and RNAz programs to provide independent predictions of fRNAs [3], [12]. These two programs should predict fRNAs independently because EvoFold utilizes a functional RNA model based on stochastic context-free grammars while RNAz primarily utilizes thermodynamic information to predict RNA secondary structures (while also considering covariance between secondary structures in a multi-species alignment). For more information about the multi-species alignments used for these fRNA prediction programs, see Text S1. The optimal multi-species alignment for each program was determined by iteratively comparing the proportion of recovered known ncRNA annotations from the SGD database [35] to the proportion of recovered known ncRNAs at a more liberal threshold (see Text S1, Tables S1,S2,S3, Figures S1,S2,S3).

The location of each fRNA was determined by the position of the middle of each fRNA secondary structure (i.e. a fRNA was in a particular category if >50% of the fold was in that type of region). All folds were categorized as coding, intronic, or intergenic.

Finally, we performed two tests to estimate potential false positive rates. First, we used the RNAfold program to calculate the minimum free energy (*mfe*) of each of the EvoFold predictions in the set of 169 genes described above [36]. If we require our folds to have a negative *mfe* for the EvoFold prediction in *S. cerevisiae*, then 148 of these genes meet this requirement and we get an estimated false positive rate of 12.4% (and every gene with a negative *mfe* in *S. cerevisiae* also has a negative average *mfe* for all the species in the multi-species alignment).

Additionally, we used the method in Katz and Burge [37]. Briefly, we calculated the excess folding potential (EFP) for genes containing coding fRNAs, as described by Katz and Burge [37]. This method uses the DicodonShuffle algorithm [37], and then uses the RNAfold program to determine if the native ORF has greater local mRNA stability than the shuffled ORF [36]. When considering the set of 169 genes considered for functional analysis (i.e. the stringently defined dataset with ribosomal genes removed), we found 101 genes containing coding fRNAs had an EFP greater than 0 (which would correspond to a 40% false positive rate). However this method may be inappropriate for our data, because we have defined in such a way that coding fRNAs must have at least a 50% overlap with coding regions, allowing folds to have some overlap with upstream and downstream regions. The codon shuffled method above cannot capture selection for stability in non-coding regions surrounding ORFs. Thus, the false positive rates estimated this method is likely an overestimate. Nevertheless, evolutionary analyses yielded similar results after excluding false positives detected by these two methods (Tables 1 and 2 versus Tables 3 and 4). This renders strong support to our conclusions that coding fRNAs likely to constrain evolutionary rates.

### GO annotation analysis

Biological Process GO Slim annotations were downloaded using the SGD GO Term Mapper interface [35], [38]. Enrichment of GO annotations was calculated by using the proportion test in R [39]. Similar results were found when a hypergeometric distribution was used to determine enrichment of GO terms. The Wilcoxon rank sum test was used to compare average values for fRNA coverage and evolutionary divergence (small dN, small dS, small dS?, small dN/dS, small dN/dS?) between the entire strictly defined dataset and subsets of genes associated with Cell Cycle (GO ID: 7049), Organelle Organization and Biogenesis (GO ID: 6996), RNA Metabolic Process (GO ID: 16070), Transcription (GO ID: 6350), Translation (GO ID: 6412), and Transport (GO ID: 6810). We used values for evolutionary divergence across a shorter timescale because they provided a larger dataset in order to control for bias due to small sample size. We choose the above subsets of genes because these are the only categories of GO annotations associated with greater than 20 genes in the strictly defined dataset, and a sample size of less than 20 genes would be too small for a robust statistical analysis.

### Calculation of nonsynonymous and synonymous divergence

We used data from Wall et al. [8] (available from the supplementary material for Drummond et al. [7]), which are evolutionary rates at synonymous and nonsynonymous sites calculated over four yeast genomes, providing an evolutionary measure of protein divergence. Additionally, we estimated divergence on the shorter timescale (referred to as small dN, small dS in the main text and Text S1) between *S. cerevisiae* and *S. paradoxus* using PAML [40]. Adjustment for codon usage bias at synonymous sites was calculated as described by [18] (namely, dS′ = dS−m* c, where m = −2.02 for the all 4-species and m = −0.386 for *S. cerevisiae*−*S. paradoxus* divergence). Recalculation on a shorter timescale is useful because it provides an opportunity to see if coding fRNAs have a different evolutionary impact on a species that are more closely related.

In order to recalculate nonsynonymous and synonymous divergence on a shorter timescale, the Multiz alignment was downloaded for all verified protein-coding genes containing at least one coding functional RNA secondary structure [34]. The Multiz alignment for these genes was obtained using the Galaxy server on the UCSC Genome Browser [41], [42]. All coding fRNAs were first defined using annotations for protein-coding genes from the SGD database [35], [42]. We only considered experimentally verified SGD annotations for protein-coding genes. In order to obtain reliable values for evolutionary divergence using PAML, the set of genes was further filtered based upon the quality of the Multiz alignment. More specifically, we removed genes with introns, premature stop codons and/or gaps in Multiz alignment, alignments without all 4 species, non-AUG start codon, and genes less than 300 bp.

### Functional variables considered in the analysis

In order to assess the biological relevance of our predicted coding fRNAs, we used rigorous statistical analysis to study the impact of fRNA coverage on evolutionary rates, relative to other previously established functional variables. The functional variables analyzed include gene expression, CAI, protein abundance, gene dispensability, gene length, degree, centrality, and mRNA half-life. Gene expression and mRNA half-life values are from Holstege et al. [43]. Codon Adaptation Index (CAI) and gene length are from Drummond et al. [9]. Protein abundance data are from Ghaemmaghami et al. [44]. Dispensability data was downloaded from http://chemogenomics.stanford.edu/supplements/01yfh/files/orfgenedata.txt [45]. The number of interactions in the yeast protein-protein interaction network (degree) was from the filtered yeast interactome data set [46]. This dataset was also used to calculate the centrality for genes in the protein-protein interaction network.

Gene length, protein abundance, degree, centrality, and mRNA half-life were excluded from certain comparisons. Gene length was excluded from analysis simply because fRNA coverage is strongly correlated with gene length, meaning that the two variables are clearly not independent. As described earlier, most other variables were excluded to remove bias from small sample size and/or overfitting.

### Multivariate statistical analysis

Partial correlation and principal component regression are two primary tools for functional genomic analysis in yeast. These statistical tools work in fundamentally different ways, and combined analysis can provide useful information about significant biological factors that govern evolutionary rates [11], [47]. More specifically, partial correlation analysis factors out the influence of a third *known* variable (or vector of known variables), while principal component regression analyzes the variance for a set of independent variables in order to identify *unknown* variables. For a more detailed discussion on the comparative performance of these two tools, see Kim and Yi [11].

Thus, principal component regression analysis requires two steps; first, a principal component analysis to define components and second, a regression analysis to determine which components have a statistically significant impact on evolutionary rates. Principal component regression was carried out using the R “pls” package [39], [48].

Partial correlation analysis can be carried out by applying the relatively straightforward equation r_DK|X_ = (r_DK_−r_DX_*r_KX_)/√[(1−r^2^_DX_)(1−r^2^_KX_)] when testing for a correlation between D and K while factoring out the influence of the third variable (or vector of variables) X. In other words, partial correlation analysis can be also used to remove the effects of a set of variables. Here, we can define X as a vector of the other N variables X_1_, X_2_, …X_N_. Then the correlation between D and K independent of X can be calculated as the correlation between D-D(X_1_, X_2_,…, X_N_) and K-K(X_1_, X_2_, …, X_N_), where D(X_1_, X_2_, …, X_N_) and K(X_1_, X_2_, …, X_N_) are the multiple linear regression of D and K, respectively, on X_1_, X_2_,..X_N_. This method was used in Kim and Yi [11] to assess the independent effect of each functional variable. We can also use the variance-covariance matrix using the assumption of normality (p. 134, [49]).

We modified R scripts available from the supplemental material for Drummond et al. 2006 for partial correlation (factoring out only expression) and principal component regression analysis [39]. An R code for our method of partial correlation analysis that controls for the influence of multiple variables (which was used to produce the data in Table 1) is available at Yi lab website (www.yilab.gatech.edu).

## Supporting Information

Text S1 (0.14 MB PDF)

Figure S1 (0.07 MB TIF)

Figure S2 (0.15 MB TIF)

Figure S3 (0.14 MB TIF)

Table S1 (0.05 MB DOC)

Table S2 (0.04 MB DOC)

Table S3 (0.05 MB DOC)

Table S4 (0.05 MB DOC)

Table S5 (0.07 MB DOC)

Table S6 (0.05 MB DOC)

Table S7 (0.05 MB DOC)
