# Supplementary material for: Predicted Functional RNAs within Coding Regions Constrain Evolutionary Rates of Yeast Proteins
Source: PLoS One. 2008 Feb 13;3(2):e1559. doi: 10.1371/journal.pone.0001559 (PMC2216430; doi:10.1371/journal.pone.0001559)
Supplement: Text S1 — (0.14 MB PDF) [file pone.0001559.s001.pdf]

Text S1.

Predicted Functional RNAs Within Coding Regions Constrain Evolutionary Rates of Yeast Proteins

Charles D. Warden[#†], Seong-Ho Kim[##], and Soojin V. Yi[#*]

[#]School of Biology
Georgia Institute of Technology
310 Ferst Drive
Atlanta, GA 30332, USA

[##]Division of Biostatistics
Indiana University School of Medicine
410 West 10th Street, Suite 3000
Indianapolis, IN 46202

†Current Address: Department of Molecular Biology, Princeton University, Princeton, NJ 08544-1014

*Corresponding author TEL 404-385-6084, FAX 404-894-2295
email: soojinyi@gatech.edu

Key Words: functional RNA, yeast comparative genomics, comparative genomics, gene ontology, partial correlation, principal component regression, evolutionary divergence

Running Head: fRNAs Constrain CDS evolution

Multi-Species Alignment: Stringent Dataset

The three types of multiple species alignments were defined as follows: 4 species – *Saccharomyces cerevisae*, *S. paradoxus*, *S. mikatae*, *S. bayanus*, 5 species – *Saccharomyces cerevisae*, *S. paradoxus*, *S. mikatae*, *S. kudriavzevii*, *S. bayanus*, 6 species - *Saccharomyces cerevisae*, *S. paradoxus*, *S. mikatae*, *S. kudriavzevii*, *S. bayanus, S. castelli*. The 4-, 5-, and 6-species alignments were based off of the Multiz alignment, which was obtained using the Galaxy server on the UCSC Genome Browser [1,2].

Before EvoFold can produce predictions of fRNA folds, genomic sequences must be pre-aligned and screened to produce small fragments because EvoFold cannot handle genomic sequences much greater than 750 basepairs in length [3]. Conserved blocks of a seven species alignment of yeast species were selected using data created by the phastCons program [4]. Only phastCons blocks containing regions of synteny described in the supplementary material from Kellis et al. [5] were selected. Initial and Final genes were determined from the file "Matches_by_chromosome_with_syn.xls" in the supplementary material from Kellis et al. [5]. Coordinates for initial and final genes were determined as described with the "*Saccharomyces cerevisae* Gene Annotation".

EvoFold predictions from the phastCons blocks were then screened using the RNAz program [6]. These two programs make predictions in fundamentally different ways (see Table S3 and Figures S1-S3). To objectively determine the optimal method to screen the original set of EvoFold predictions, the proportion of known annotations recovered for a particular method was compared to the number of folds retained by imposing a more "strict" significance level (although it was unclear if the FPS values for the EvoFold program really corresponded to more accurate predictions – see EvoFold Program section for more details). For example, increasing the RNAz p-value from 0.5 to 0.9 reduced the total number of folds to 38% of the original total number of folds but still retained 73% percent of known tRNAs, 60% of snoRNAs, 60% of snRNAs, 100% of rRNAs, and 93% of miscellaneous RNAs predicted by the RNAz program for the 5-species alignment at the p-value of 0.5. The most accurate dataset was the set of EvoFold predictions produced by the 5-species alignment (with an FPS value greater than 0) that were independently verified by the RNAz predictions made using the 6-species alignment with an RNAz p-value of 0.9. An EvoFold prediction was considered to be independently verified by the RNAz program if the middle of the EvoFold prediction was within an RNAz prediction (so that at there would be at least 50% overlap between

the two predictions). Furthermore, all predictions used in the stringent dataset were also required to be greater than 10 nucleotides in length.

EvoFold Program

The EvoFold program was used to predict fRNA secondary structures in post-WGD species of yeast, and it took approximately one month to compete a whole genome screening with four species alignment (*Saccharomyces cerevisae*, *S. paradoxus*, *S. mikatae*, and *S. baynus*), as described in the “Multi-Species Alignment” section. Using a Linux cluster (6 AMD Opeteron nodes – 2 dual processor, dual core), all other comparisons took approximately one week.

The significance of the fold level was determined by a folding potential score (FPS). FPS is a length normalized likelihood-ratio score and is defined as follows: FPS = $\log (P(x|\phi_{fRNA})/P(x|\phi_{bg})) / l$, where $P(x|\phi_{fRNA})$ refers to the probability that a sequence fits an fRNA structural model, $P(x|\phi_{bg})$ refers to the probability that the sequence fits the background model (i.e. no-fRNA structure model), and l refers to the length of the fold (defined by the outermost basepair of a fRNA structure) [3].

We required all folds in the final dataset to have an FPS greater than 0. Although this may not seem like a very stringent requirement, this requirement decreases the sample size of EvoFold predictions by close to 50% (see Figure S3). The average FPS for the folds predicted using the 5-species alignment was only 0.136 with a standard deviation of 0.609. It should also be noted that FPS is not symmetrically distributed - there are a small number of folds in the 5-species EvoFold dataset that have an FPS greater than 3, but there no folds with an FPS smaller than -3. Therefore, the average for the 5-species alignment is biased towards larger values. This is also demonstrated by the observation that the median FPS, 0.055, is clearly much smaller than the mean value for this dataset. Using a higher cutoff value for the FPS score does not substantially increase the accuracy of our dataset because imposing a higher FPS cutoff does not increase the proportion of recovered positive controls (possibly due to the sharp decrease in sample size).

RNAz Program

Unlike the EvoFold program, the RNAz program relies mostly on thermodynamic information to predict RNA secondary structures [6]. However, this program also utilizes the same 4-, 5-, and 6- species alignments from the phastCons blocks that were used

for the EvoFold predictions, and the program gives a “bonus” decrease in minimum free energy (MFE) of covariance between each of the yeast sequences and the consensus sequence (and poorly conserved secondary structures are likewise given an increase in the minimum in the overall MFE value). RNAz gives a p-value for its predictions, but this p-value is not equivalent to the traditional statistical definition of a p-value because there is *no underlying statistical model*. Although RNAz utilizes an SCI-value to measure structural conservation between aligned sequences and a z-score to measure thermodynamic stability, the p-value provides the user a more comprehensive value for final classification. More specifically, RNAz uses an *ad hoc* machine learning technique to produce p-values based on estimated false positive rates [6]. For example, the RNAz manual states that a p-value > 0.5 should result in an approximately 4% false positive rate while a p-value of 0.9 should result in false positive rate of ~ 1%. However, these estimations are based on an *artificially* designed background set of data, so the actual false positive rate will vary for different datasets.

Nevertheless, increasing p-values led to increased recovery of positive controls (see Table S3 and Figure S2). However, it should also be noted that RNAz recovers a larger proportion of RNA secondary structures within coding regions, so the requirement of independent verification by RNAz and EvoFold will be more conservative for intergenic than coding fRNAs.

Saccharomyces cerevisae Gene Annotation

Whenever possible, coordinates for genes were based upon the “SGD Genes” table for the *Saccharomyces cerevisae* genome sequence available on the UCSC Genome Browser [2]. Unless otherwise noted in Table S2, all coordinates for genes came from the “SGD Genes” table. If a gene name could not be found within this table, data available from the “SGD Other” table from the UCSC Genome Browser was used [2]. In the event that the gene annotation could still not be found, coordinates from the SGD_features.tab file available from the FTP for the *Saccharomyces* Genome Database were used for that particular gene [7].

Supplementary Figure Legends

Figure S1. Total Number of Folds Predicted by Various Methods.

Figure S2. Distribution of predicted fRNAs from the EvoFold algorithm. The X axis depicts three different alignments used for prediction. The Y-axis depicts the number of folds, color-coded for different genomic regions.

Figure S3. Distribution of predicted fRNAs from the RNAz algorithm. The X axis depicts different alignments and cutoff values. The Y-axis depicts the number of folds, color-coded for different genomic regions.

Bibliography

1. Giardine B, Riemer C, Hardison RC, Burhans R, Elnitski L, et al. (2005) Galaxy: A platform for interactive large-scale genome analysis. Genome Research 15: 1451-1455.
2. Karolchik D, Baertsch R, Diekhans M, Furey TS, Hinrichs A, et al. (2003) The UCSC Genome Browser Database. Nucleic Acids Research 31: 51-54.
3. Pedersen JS, Bejerano G, Siepel A, Rosenbloom K, Lindblad-Toh K, et al. (2006) Identification and classification of conserved RNA secondary structures in the human genome. PLoS Computational Biology 2: 251-262.
4. Siepel A, Bejerano G, Pedersen JS, Hinrichs AS, Hou MM, et al. (2005) Evolutionarily conserved elements in vertebrate, insect, worm, and yeast genomes. Genome Research 15: 1034-1050.
5. Kellis M, Birren BW, Lander ES (2004) Proof and evolutionary analysis of ancient genome duplication in the yeast Saccharomyces cerevisiae. Nature 428: 617-624.
6. Washietl S, Hofacker IL, Stadler PF (2005) Fast and reliable prediction of noncoding RNAs. Proceedings of the National Academy of Sciences of the United States of America 102: 2454-2459.
7. Cherry JM, Ball C, Weng S, Juvik G, Schmidt R, et al. (1997) Genetic and physical maps of Saccharomyces cerevisiae. Nature 387: 67-73.
