# Supplementary figures and images for: Predicted Functional RNAs within Coding Regions Constrain Evolutionary Rates of Yeast Proteins
Source: PLoS One. 2008 Feb 13;3(2):e1559. doi: 10.1371/journal.pone.0001559 (PMC2216430; doi:10.1371/journal.pone.0001559)

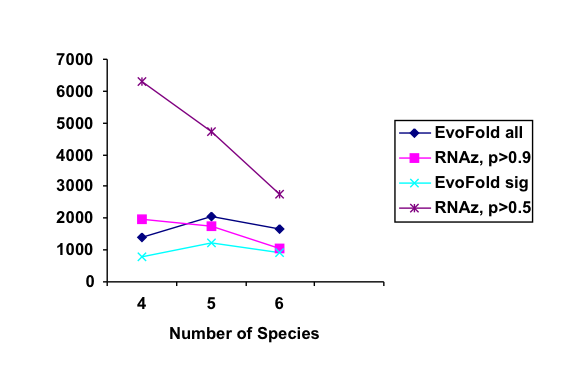

Supplement: Figure S1 — (0.07 MB TIF) [file pone.0001559.s002.tif]

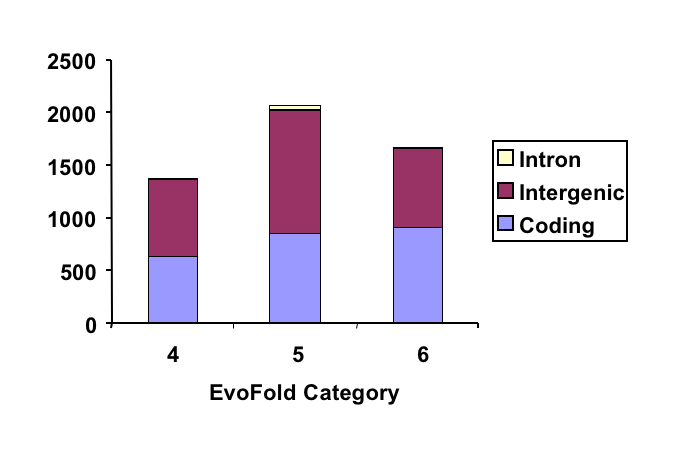

Supplement: Figure S2 — (0.15 MB TIF) [file pone.0001559.s003.tif]

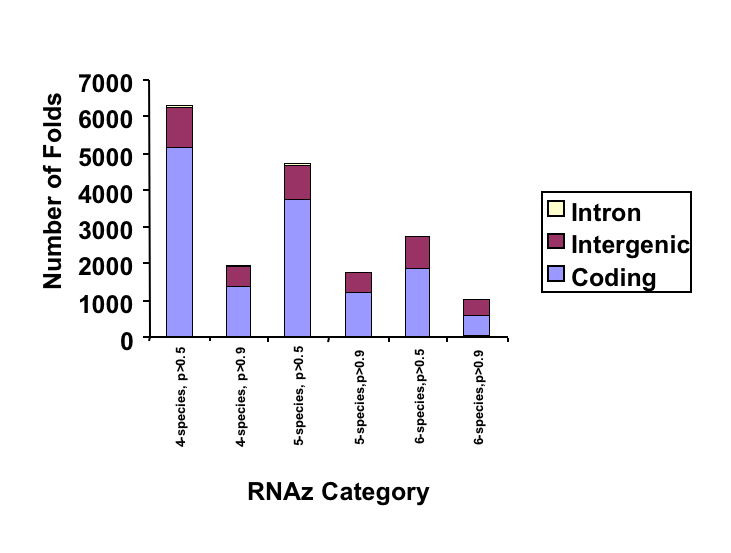

Supplement: Figure S3 — (0.14 MB TIF) [file pone.0001559.s004.tif]
