# Supplementary material for: Predicted Functional RNAs within Coding Regions Constrain Evolutionary Rates of Yeast Proteins
Source: PLoS One. 2008 Feb 13;3(2):e1559. doi: 10.1371/journal.pone.0001559 (PMC2216430; doi:10.1371/journal.pone.0001559)
Supplement: Table S1 — (0.05 MB DOC) [file pone.0001559.s005.doc]

Table S1: Recovered Annotations using Various Prediction Methods

| **Prediction Category** | **tRNA**  **(out of 275)** | **rRNA**  **(out of 11)** | **snoRNA (out of 66)** | **snRNA (out of 6)** | **Misc RNA (out of 14)** |
| --- | --- | --- | --- | --- | --- |
| **EvoFold 4 all** | 34 | 7 | 21 | 4 | 11 |
| **EvoFold 4 FPS > 0** | 25 | 5 | 14 | 2 | 6 |
| **EvoFold 5 all** | 63 | 7 | 28 | 5 | 11 |
| **EvoFold 5 FPS > 0** | 45 | 6 | 22 | 5 | 11 |
| **EvoFold 6 all** | 85 | 9 | 18 | 5 | 8 |
| **EvoFold 6 FPS > 0** | 77 | 13 | 2 | 8 | 6 |
| **RNAz 4 p>0.5** | 184 | 8 | 48 | 6 | 14 |
| **RNAz 4 p>0.9** | 112 | 7 | 32 | 3 | 12 |
| **RNAz 5 p>0.5** | 163 | 7 | 45 | 5 | 14 |
| **RNAz 5 p>0.9** | 119 | 7 | 27 | 3 | 13 |
| **RNAz 6 p> 0.5** | 150 | 7 | 31 | 4 | 14 |
| **RNAz 6 p>0.9** | 107 | 7 | 25 | 4 | 12 |

Number in 1^st^ column (e.g. 4, 5, or 6) refers to the number of species used in multi-species alignment. FPS stands for folding potential score.
