# Supplementary material for: Predicted Functional RNAs within Coding Regions Constrain Evolutionary Rates of Yeast Proteins
Source: PLoS One. 2008 Feb 13;3(2):e1559. doi: 10.1371/journal.pone.0001559 (PMC2216430; doi:10.1371/journal.pone.0001559)
Supplement: Table S2 — (0.04 MB DOC) [file pone.0001559.s006.doc]

Table S2: fRNA coverage provides more information than 1/ length alone:

|  | **dN** | **dS** | **dS´** | **dN/dS** | **dN/dS´** |
| --- | --- | --- | --- | --- | --- |
| **fRNA coverage|_len_^1^** | -0.247* | -0.200^#^ | -0.203* | -0.205* | -0.231* |
| **fRNA coverage^2^** | -0.299** | -0.348*** | -0.403**** | -0.209* | -0.261* |
| **1/length^3^** | -0.194^#^ | -0.291** | -0.362*** | -0.110 | -0.158 |

The table above demonstrates that the variable fRNA coverage has a significant influence on evolutionary rates independent of the variable gene length alone.

^1^The first row is the partial correlation analysis between fRNA coverage and evolutionary rates after controlling for gene length.

^2^The second row is the correlation between fRNA coverage and evolutionary rates. The data set used for this correlation is those with all 7 functional variables available.

^3^The third row is the correlation between 1/gene length and evolutionary rates, for the same data set. We can see that fRNA coverage explains greater amount of variance than gene length alone.

p-values: #=0.1, *=0.05, **=0.01, ***=0.001, ****=10^-4^
