# Supplementary material for: Predicted Functional RNAs within Coding Regions Constrain Evolutionary Rates of Yeast Proteins
Source: PLoS One. 2008 Feb 13;3(2):e1559. doi: 10.1371/journal.pone.0001559 (PMC2216430; doi:10.1371/journal.pone.0001559)
Supplement: Table S3 — (0.05 MB DOC) [file pone.0001559.s007.doc]

**Table S3: Correlations and Partial Correlations using Pearson Correlations with**

Additional Functional Variables

|  | dN | dS | dS´ | dN/dS | dN/dS´ |
| --- | --- | --- | --- | --- | --- |
| Gene Expression | -0.244  (-0.656***) | -0.213  (-0.751****) | -0.241  (-0.240) | -0.194  (-0.515*) | -0.207  (-0.641**) |
| CAI | -0.326  (-0.613**) | -0.539  (-0.716***) | 0.178  (0.108) | -0.199  (-0.477*) | -0.368  (-0.640**) |
| Dispensability | 0.257  (0.119) | -0.326  (-0.117) | -0.289  (-0.047) | 0.317  (0.176) | 0.298  (0.126) |
| Degree | 0.374  (0.033) | -0.065  (-0.173) | -0.159  (-0.104) | 0.378  (0.096) | 0.395  (0.045) |
| Protein Centrality | -0.158  (-0.176) | -0.068  (-0.254) | -0.107  (-0.051) | -0.134  (-0.117) | -0.130  (-0.170) |
| **fRNA Coverage** | -0.208  (-0.190) | **-0.551***  (-0.219) | **-0.428^#^**  **(-0.556**)** | -0.069  (-0.150) | **-**0.157  (-0.128) |
| mRNA Half-life | 0.211  (0.290) | -0.247  (0.111) | -0.221  (-0.006) | 0.261  (0.301) | 0.258  (0.301) |

Note: Pearson Correlations are shown in parenthesis below partial correlation in the above table. Ribosomal genes are removed and all other factors are considered for partial correlation analysis. Sample size = 22 genes; p-values: #=0.1, *=0.05, **=0.01, ***=0.001, ****=10^-4^.
