# Supplementary material for: Predicted Functional RNAs within Coding Regions Constrain Evolutionary Rates of Yeast Proteins
Source: PLoS One. 2008 Feb 13;3(2):e1559. doi: 10.1371/journal.pone.0001559 (PMC2216430; doi:10.1371/journal.pone.0001559)
Supplement: Table S4 — (0.05 MB DOC) [file pone.0001559.s008.doc]

**Table S4: Correlations and Partial Correlations using Pearson Correlations for a**

Shorter Evolutionary Timescale

|  | Small dN | Small dS | Small dS´ | Small dN/dS | Small dN/dS´ |
| --- | --- | --- | --- | --- | --- |
| Gene Expression | 0.148^#^  (-0.479****) | -0.203*  (-0.451****) | -0.161^#^  (-0.062) | -0.092  (-0.343****) | -0.122  (-0.458****) |
| CAI | -0.240**  (-0.495****) | -0.098  (-0.392****) | 0.248**  (0.113) | -0.169^#^  (-0.361****) | -0.262**  (-0.495***) |
| Dispensability | 0.144  (0.245) | 0.265**  (0.346****) | -0.245**  (0.243**) | 0.069  (0.152^#^) | 0.093  (0.199*) |
| **fRNA Coverage** | -0.078  (**-0.192***) | -0.080  (**-0.185***) | -0.102  **(**-0.107**)** | -0.071  (-0.156^#^) | -0.073  (-0.183*) |

Note: Pearson Correlations are shown in parenthesis below partial correlation in the above table. For above dataset, ribosomal genes are removed and all other factors are considered for partial correlation analysis. Sample size is 128 genes.

Significant correlations with fRNA coverage are shown in bold; p-values: #=0.1, *=0.05, **=0.01, ***=0.001, ****=10^-4^.

Evolutionary rates beginning with “small” represent divergence only between *S. cerevisiae* and *S. paradoxus* whereas all other evolutionary rates represent values from Wall et al. 2005 (summed pairwise divergence between *S. cerevisiae*, *S. paradoxus*, *S. mikatae*, and *S. bayanus*).
