# Supplementary material for: Predicted Functional RNAs within Coding Regions Constrain Evolutionary Rates of Yeast Proteins
Source: PLoS One. 2008 Feb 13;3(2):e1559. doi: 10.1371/journal.pone.0001559 (PMC2216430; doi:10.1371/journal.pone.0001559)
Supplement: Table S5 — (0.07 MB DOC) [file pone.0001559.s009.doc]

**Table S5: Results of Principal Component Regression Analysis with Additional**

Functional Variables

|  | Principal Components | | | | | | | |
| --- | --- | --- | --- | --- | --- | --- | --- | --- |
|  | 1 | 2 | 3 | 4 | 5 | 6 | 7 | All |
| Component Composition:^1^ |  |  |  |  |  |  |  |  |
| Gene Expression | 0.165 | 0.074 | **0.371** | 0.001 | 0.020 | 0.027 | **0.341** |  |
| CAI | **0.284** | 0.188 | 0.029 | 0.003 | 0.016 | 0.064 | **0.416** |  |
| Dispensability | 0.000 | 0.198 | 0.127 | **0.383** | **0.223** | 0.066 | 0.003 |  |
| Degree | 0.199 | 0.073 | 0.061 | 0.185 | 0.088 | **0.373** | 0.020 |  |
| Protein Centrality | **0.234** | 0.054 | 0.177 | 0.003 | 0.045 | **0.379** | 0.108 |  |
| **fRNA Coverage** | 0.001 | **0.295** | **0.232** | 0.097 | **0.247** | 0.016 | 0.112 |  |
| mRNA Half-life | 0.116 | 0.118 | 0.002 | **0.327** | **0.360** | 0.076 | 0.000 |  |
|  |  |  |  |  |  |  |  |  |
| Percent Variance Explained:^2^ |  |  |  |  |  |  |  |  |
| dN | **26.87** | 1.67 | **24.11** | 2.81 | 1.46 | 5.8 | 0.05 | **62.77** |
| dS | **39.3** | 6.26 | **18.37** | 1.81 | **7.89** | 1.46 | 2.03 | **77.12** |
| dS´ | 0.41 | 6.1 | 8.50 | 2.97 | **25.46** | 0.4 | 0.57 | **44.42** |
| dN/dS | 15.62 | 0.47 | **18.68** | 2.31 | 5.43 | 5.62 | 0.03 | **48.16** |
| dN/dS´ | **27.29** | 2.6 | **21.86** | 2.37 | 3.46 | 6.07 | 0.17 | **63.81** |
|  |  |  |  |  |  |  |  |  |

**^1^**Numbers in bold correspond to predictors that contribute at least 20% to indicated component.

**^2^**Using information from regression analysis, underlined font means p-values < 0.1; bold font means p-value < 0.05

Sample size is 22 genes.
