# Supplementary material for: Predicted Functional RNAs within Coding Regions Constrain Evolutionary Rates of Yeast Proteins
Source: PLoS One. 2008 Feb 13;3(2):e1559. doi: 10.1371/journal.pone.0001559 (PMC2216430; doi:10.1371/journal.pone.0001559)
Supplement: Table S6 — (0.05 MB DOC) [file pone.0001559.s010.doc]

**Table S6: Results of Principal Component Regression Analysis for a Shorter**

Evolutionary Timescale

|  | Principal Components | | | | |
| --- | --- | --- | --- | --- | --- |
|  | 1 | 2 | 3 | 4 | All |
| Component Composition:^1^ |  |  |  |  |  |
| Gene Expression | **0.413** | 0.000 | 0.060 | **0.527** |  |
| CAI | **0.380** | 0.000 | 0.158 | **0.462** |  |
| Gene Dispensability | 0.102 | **0.511** | **0.381** | 0.005 |  |
| **fRNA Coverage** | 0.105 | **0.489** | **0.401** | 0.005 |  |
|  |  |  |  |  |  |
| Percent Variance Explained:^2^ |  |  |  |  |  |
| Small dN | **28.63** | 0.17 | 0.21 | 0.18 | **29.18** |
| Small dS | **24.86** | 1.44 | 0.51 | 0.20 | **27.01** |
| Small dS´ | 0.34 | 1.05 | **7.39** | **3.77** | **12.56** |
| Small dN/dS | **14.84** | 0.00 | 0.15 | 0.14 | **15.12** |
| Small dN/dS´ | **26.34** | 0.02 | 0.59 | 0.41 | **27.36** |
|  |  |  |  |  |  |

**^1^**Numbers in bold correspond to predictors that contribute at least 20% to indicated component. Sample size is 128 genes.

**^2^**Using information from regression analysis, underlined font means p-values < 0.1; bold font means p-value < 0.05
