# Supplementary material for: Predicted Functional RNAs within Coding Regions Constrain Evolutionary Rates of Yeast Proteins
Source: PLoS One. 2008 Feb 13;3(2):e1559. doi: 10.1371/journal.pone.0001559 (PMC2216430; doi:10.1371/journal.pone.0001559)
Supplement: Table S7 — (0.05 MB DOC) [file pone.0001559.s011.doc]

**Table S7: Short versus Long Genes:**

A. Correlation Analysis

|  | **Whole Data** | **No Ribosomal genes** |
| --- | --- | --- |
| **dN ~ cov** | -0.361**** | -0.299* |
| **dS´ ~ cov** | -0.398**** | -0.403**** |
| **Small dN ~ cov** | -0.279*** | -0.170* |
| **Small dS ~ cov** | -0.407**** | -0.180* |
| **dN ~ FPS** | -0.270** | -0.134 |
| **dS´ ~ FPS** | -0.198* | -0.125 |
| **Small dN ~ FPS** | -0.221** | -0.101 |
| **Small dS ~ FPS** | -0.354**** | -0.131 |

All values are log normalized. For p-values, *=0.05, **=0.01, ***=0.001, **** =0.0001. cov = fRNA coverage. Small = sacCer/sacPar dN or dS.

**B. Average Evolutionary Rate Analysis**

| **Comparison (short, long)** | **p-value (15 nt cutoff)** | **p-value (20 nt cutoff)** |
| --- | --- | --- |
| dN | 0.135 (-1.03, -1.17) | 0.318 (-1.10, -1.19) |
| dS´ | 0.471 (0.313, 0.320) | 0.866 (0.318,0.320) |
| Small dN | 0.339 (-1.68, -1.75) | 0.896 (-1.75, -1.76) |
| Small dS | 0.603 (-0.483, -0.502) | 0.886 (-0.509,-0.514) |
| FPS | 0.888 (-1.02, -1.00) | 0.802 (-0.998, -0.979) |

All values are log normalized. Means are shown in parentheses (short, long)
